# The clinical and neuroimaging differences between vascular parkinsonism and Parkinson’s disease: a case-control study

**DOI:** 10.1186/s12883-024-03556-9

**Published:** 2024-02-06

**Authors:** Peter George, Tamer Roushdy, Mai Fathy, Eman Hamid, Yosra Abdelzaher Ibrahim, Mahmoud El-Belkimy, Mohamed Ossama Abdulghani, Ali Shalash

**Affiliations:** 1 Department of Neurology, Faculty of Medicine, Ain Shams University, Cairo, Egypt; 2 Department of Radiology, Faculty of Medicine, Ain Shams University, Cairo, Egypt

**Keywords:** Parkinson’s disease, Vascular parkinsonism, Non-motor, TCCD, Brain MRI, Quality of life

## Abstract

**Background:**

Parkinson’s disease (PD) and vascular parkinsonism (VaP) have highly overlapping phenotypes, and different prognosis. This study comprehensively investigated the clinical, brain MRI and transcranial sonography differences between VaP and PD.

**Methods:**

Forty-eight patients with PD, 27 patients with VaP, and 29 healthy controls were compared. All patients were assessed using the MDS-UPDRS, Berg Balance Scale (BBS), Ten-Meter Walking Test (10-MWT), Time Up and Go Test, and Non-Motor Symptoms Scale. Beck Depression Inventory, PD questionnaire- 39, international urine incontinence scale, cognitive assessment scales, MRI brain and transcranial colour-coded doppler. The study was registered on clinical-Trial.gov (NCT04308135) on 03/12/2020.

**Results:**

VaP patients showed significantly older age of onset, shorter disease duration, lower drug doses and levodopa responsiveness, higher On and Off axial scores, On and Off BBS, higher On scores for PIGD, rigidity, bradykinesia and total motor MDS-UPDRS, lower On and Off tremor, lower-half predominance, lower asymmetrical presentation and symmetric index than PD patients. VaP patients had worse non-motor symptoms Scale (NMSS) than controls except for perceptual problems/hallucinations but better symptoms than PD patients except for urinary dysfunction. Quality of life (QoL) was impaired in VaP patients and was correlated with motor function and NMSs. The VaP group had significantly higher white matter lesions and brain atrophy, with lower hyperechogenicity of the substantia nigra and more impaired cerebral vascular resistance and vasoreactivity than the PD group.

**Conclusions:**

VaP has a characteristic motor and non-motor profile, with impaired QoL, white matter, and transcranial sonography abnormalities that differentiate it from PD. Further studies are warranted to explore the role of vascular lesions in the pathogenesis of VaP.

**Trial registration:**

The registered identifier NCT04308135 on clinical-Trial.gov. Registered on 03/12/2020.

**Supplementary Information:**

The online version contains supplementary material available at 10.1186/s12883-024-03556-9.

## Background

Parkinson’s disease (PD) and vascular parkinsonism (VaP) have highly overlapping phenotypes, with mixed pathologies, making distinguishing between these two parkinsonian diseases challenging [1]. VaP is a form of acquired parkinsonism in which the parkinsonian features are of vascular origin in contrast to PD, which is neurodegenerative in etiology [2]. It accounts for 4.4–12% of all cases of parkinsonism and is associated with vascular changes in the globus pallidus, white matter, and to a lesser extent, in the substantia nigra (SN) [3]. Consequently, common risk factors for VaP are the same as those for cerebrovascular disease, and their prevention and treatment are of utmost importance [1]. 

Few studies have identified the characteristic clinical features of VaP compared to PD [4]. Additionally, investigations such as magnetic resonance imaging (MRI) and transcranial color-coded Doppler (TCCD) could aid in differentiating VaP from PD. TCCD was found to display increased hyperechogenicity at the SN in patients with PD compared to normal controls or participants with other Parkinsonian syndromes [5]. 

However, VaP, particularly the insidious form, is still a debatable concept due to the lack of correlation between vascular risk factors, white matter lesions (WMLs) and parkinsonian features and its overlap with high-order gait disorder, PD or atypical parkinsonism [6]. Therefore, differentiating PD and VaP is clinically important due to the overlapping clinical characteristics as well as different responses to dopaminergic drugs and prognoses [7]. Moreover, identifying clinical and neuroimaging differences between the two diseases is essential to characterize and justify the concept of VaP.

The aim of the current study was to comprehensively investigate the differences between VaP and PD, including clinical profile (motor, non-motor symptoms (NMSs), and gait), radiological and transcranial sonographic characteristics (MRI brain, carotid duplex and TCCD) and laboratory tests.

## Methods

This case–control study compared age and gender matched patients with PD, patients with VaP and healthy controls. Consecutive patients were recruited from movement disorders and stroke outpatient clinics at Ain Shams University Hospitals during the period from March 2020 to December 2021. Age and gender healthy controls were recruited from other patients’ companions and relatives visiting the hospital. The study was approved by the ethical committee of the Faculty of Medicine, Ain Shams University, and was registered on clinical-Trial.gov (NCT04308135) on March 12, 2020. Written informed consent was obtained from all participants. In a one-way ANOVA study, sample sizes of at least 40 PD patients, 24 VaP patients, and 24 controls were obtained representing the 3 groups whose means are to be compared. The total sample of 88 subjects achieves 80% power to detect differences among the means versus the alternative of equal means using an F test with a 0.05 significance level.

### Motor and non-motor assessments

The diagnosis of PD was based on the Movement Disorders Society (MDS) diagnostic criteria [8], while the diagnosis of VaP was based on the Zijlmans et al. criteria for probable VaP [9]. Patients were excluded if they had any alternative cause that significantly impairs gait, had any contraindication for neuroimaging, had a poor transtemporal window in TCCD or could not perform the tests. Patients with atypical and other causes of secondary parkinsonism were also excluded.

All patients were subjected to a comprehensive clinical evaluation and laboratory investigations and were assessed in OFF and ON states using the MDS-Unified Parkinson disease rating scale (MDS-UPDRS), modified Hoehn and Yahr scale (H&Y), Schwab and England activities of daily living scale (S&E-ADL) [10, 11], new freezing of gait questionnaire (NFOG-Q) [12], 10-metre walk test [13], time up and go (TUG) test [14], and Berg balance scale [15]. The lower body predominance was determined by a two-point difference between the upper and lower limb scores of bradykinesia, rigidity or postural instability of the MDS-UPDRS-III [4]. Clinical asymmetry was defined as the difference between the summed MDS-UPDRS scores of the left and right extremities (items 3.3–3.8 and 3.15–3.17) [16]. The symmetric index parameter was calculated for asymmetrical subjects. Higher values indicate higher degrees of asymmetry [17]. The motor subtypes were determined for the PD and VaP groups [18]. Using the dopa challenge test, the proper response to levodopa was considered if the MDS-UPDRS-III improved by more than 24.5% [19]. For patients with VaP who are mostly drug naïve, we used a morning dosage of Levodopa/carbidopa 250/25 mg preceded by domperidone (10 mg). Patients with PD, who were under treatment, were received 120% of the morning levodopa dose [20]. 

All patients and controls were evaluated by the non-motor symptoms scale (NMSS) for NMSs [21], the Beck depression inventory (BDI) [22], Parkinson’s Disease Questionnaire (PDQ-39) for quality of life (QoL) [23], the Arabic version of Montreal Cognitive Assessment (MoCA) [24], the Wechsler memory scale-III (WMS) [25], Frontal Assessment Battery (FAB) for executive functions [26], the verbal fluency test, and the clock drawing test (for visuospatial skills) of Addenbrooke’s cognitive examination-III (ACE-III) [27]. Patients’ urinary symptoms were assessed by the Arabic version of the International Consultation on Incontinence Questionnaire-Short Form (ICIQ-SF) [28]. 

### MRI brain assessment

Brain MRI was performed for all patients using a 1.5-T Siemens Magnetom Symphony scanner machine for assessing the WMLs by the Fazekas scale [29] and the Scheltens scale [30] and assessing brain atrophy using the Scheltens-Graz visual rating scale [31]. MRI brain included T1-weighted imaging, T2-weighted imaging, diffusion-weighted imaging, and fluid-attenuated inversion recovery.

### Vascular and substania nigra ultrasound assessments

TCCD (Esaote My Lab Five, Italy) was performed using a color-coded ultrasound system with a phase array 2 Hz probe through the transtemporal bone window. The mean flow velocity (MFV) and pulsatility index (PI) of the middle and posterior cerebral arteries (MCA, PCA) were recorded bilaterally [32]. Cerebral vasomotor reactivity (CVR) was evaluated by measuring the breath holding index (BHI) [33]. The hyperechogenic area at the SN was measured automatically by encircling the outer circumference of the area of ​​hyperechogenicity, and the highest value was recorded [34]. Patients with at least one echogenic size ≥ 18 mm^2^ were classified as hyperechogenic [35]. The ultrasonographic examination of extracranial vessels was performed using a 12 Hz linear probe (Esaote My Lab Five, Italy), measuring the intimal medial thickness (IMT) of the common carotid artery (CCA) in B-mode. The carotid arteries were evaluated for the presence of atherosclerotic plaques, the degree of stenosis, and the peak systolic velocity (PSV) [36]. Controls underwent TCCD and ultrasonographic examination of extracranial vessels.

### Statistical analysis

Data analysis was performed using IBM SPSS software package version 25.0 (Armonk, NY: IBM Corp). The Mann‒Whitney test was used for nonnormally distributed quantitative variables, and Chi-square test was applied to assess the statistical significance between categorical variables. The Kruskal‒Wallis test was used to assess the statistical significance of the difference between more than two study groups. Correlation analyses between the variables were performed by Spearman correlation coefficients. A *p* value of 0.05 or less was considered statistically significant, except in the case of correlation; after using Bonferroni correction, it became < 0.004.

## Results

A total of 104 participants, 48 PD patients, 27 VaP patients and 29 healthy controls, were included in the study. Eight patients were excluded, including a patient with frontal meningioma, one with exposure to antipsychotic drugs, one with progressive supranuclear palsy, two patients with normal pressure hydrocephalus (NPH) and three patients with severe knee osteoarthritis causing gait impairment.

The mean age of PD group was 58.6 ± 6.2 years, while the mean age of VaP group was 63.1 ± 10.3 years. The three groups were matched regarding age, gender, and years of education. Vascular risk factors were significantly more frequent among VaP group, compared to PD group (hypertension, hyperlipidaemia, smoking (*p* < 0.001), diabetes mellitus (*p* = 0.018), ischemic heart disease (*p* = 0.003)), while consanguinity and family history of PD were significantly more frequent among patients with PD (*p* < 0.001 and 0.025, respectively) (Table 1).

**Table 1:** Demographics and clinical characteristics of patients with vascular parkinsonism, Parkinson’s disease, and controls

		Parkinson’s disease (No. = 48)	Vascular parkinsonism (No. = 27)	Control (No. = 29)	Chi-square test
		Frequency (%)	Frequency (%)	Frequency (%)	***P*** value
**Age (Mean /SD) ^**		58.6 (6.2)	63.1 (10.3)	58.86 (8.2)	0.054
**Gender** ***(Male/female)***		36 / 12 (75% / 25%)	21 / 6 (77.8%/ 22.2%)	16 / 13 (55.2%/ 44.8%)	0.111
**Functioning**	**Non-functioning**	5 (10.4%)	5 (18.5%)	0 (0.0%)	0.027*
	**Functioning**	29 (60.4%)	8 (29.6%)	16 (55.2%)	
	**Retired**	14 (29.2%)	14 (51.9%)	13 (44.8%)	
**Number of vascular risk factors**	**0**	26 (54.2%)	0 (0.0%)	29 (100%)	< 0.001*
	**1**	14 (29.2%)	1 (3.7%)	0 (0.0%)	
	**2**	6 (12.5%)	15 (55.6%)	0 (0.0%)	
	**3**	2 (4.2%)	8 (29.6%)	0 (0.0%)	
	**4**	0 (0.0%)	3 (11.1%)	0 (0.0%)	
**Smoking**	**Non-smoker**	32 (66.7%)	11 (40.7%)	25 (86.2%)	< 0.001*
	**Smoker**	6 (12.5%)	16 (59.3%)	4 (13.8%)	
	**Ex-smoker**	10 (20.8%)	0 (0.0%)	0 (0.0%)	
**Years of education** ^**τ**^ ***(median/range)***		6 (0–18)	8 (0–13)	7 (0–16)	0.613
**Substance abuse**		2 (4.2%)	0 (0.0%)	0 (0.0%)	0.304
**Diabetes mellitus**		9 (18.8%)	7 (25.9%)	0 (0.0%)	0.018*
**Hypertension**		13 (27.1%)	25 (92.6%)	0 (0.0%)	< 0.001*
**Hepatitis C virus**		1 (2.1%)	0 (0.0%)	0 (0.0%)	0.555
**Ischemic heart disease**		8 (16.7%)	9 (33.3%)	0 (0.0%)	0.003*
**Hyperlipidemia**		2 (4.2%)	27 (100.0%)	0 (0.0%)	< 0.001*
**Consanguinity**		14 (29.2%)	1 (3.7%)	0 (0.0%)	< 0.001*
**Family History of Neuropsychiatric Illness**	**No**	35 (72.9%)	26 (96.3%)	29 (100.0%)	0.025*
	**Parkinson’s disease**	12 (25%)	1 (3.7%)	0 (0.0%)	
	**Psychiatric**	1 (2.1%)	0 (0.0%)	0 (0.0%)	
	**Dementia**	1 (2.1%)	0 (0.0%)	0 (0.0%)	

^One Way ANOVA test is used

^τ^ Kruskal-Wallis Test is used

*p value is significant

### Motor characteristics of PD and VaP

Compared to PD group, VaP group showed significantly older age of onset (*p* < 0.001), shorter disease duration (the time from the onset of motor symptoms to the time of evaluation) (*p* = 0.007), lower-half predominance (*p* = 0.005), worse MDS-UPDRS-III On (*p* = 0.002), rigidity On (*p* < 0.001), bradykinesia On (*p* = 0.035), worse On and Off axial scores (*p* < 0.001 and 0.028, respectively), PIGD On scores (*p* < 0.001), On and Off BBS (*p* < 0.001), TUG-On (*p* = 0.003), and ICIQ-SF (*p* = 0.014), while lower tremor On and Off (*p* < 0.001 and 0.007, respectively), asymmetrical presentation and symmetric index (*p* < 0.001), NFOG-Q-OFF (*p* < 0.010), levodopa equivalent daily dose (LEDD) (*p* < 0.001) and percentage of levodopa responsiveness (*p* < 0.001). Seven patients with VaP (25.9%) showed response to levodopa, with mean LEDD was 467.86 mg (range 425 to 525 mg). Seventeen (63%) patients with VaP had insidious presentation, while 10 had acute presentation (37%). Patients with VaP were more frequently of the PIGD type (16 (59.3%)) than patients with PD (6 (12.5%)), who more frequently had the TD type (37 (77.1%)) (*p* > 0.001) (Table 2). Twenty-four (88.9%) patients with VaP had pyramidal signs. There were no significant differences in MDS-UPDRS-I, II, IV and total scores, dyskinesia, part III-Off, rigidity, bradykinesia, PIGD-Off and TUG-Off between the groups (Table 2).

**Table 2:** comparison of motor characteristics between Parkinson’s disease and vascular parkinsonism

		Parkinson’s disease (No. =48)	Vascular parkinsonism (No. =27)	Mann Whitney U test / Chi-square test^	
		Median (IQR/Range)	Median (IQR/Range)	z/ x	***p***
AOO (years)		53.25(40–74)	65 (41–71)	-3.552	< 0.001*
DOI (years)		5 (1.50–14)	3 (1–10)	-2.693	0.007*
Onset *^*	Gradual	48 (100%)	17 (63%)		
	Acute	0 (0%)	10 (37%)		
Motor subtypes	Tremor dominant	37 (77.1%)	7 (25.9%)	20.867	< 0.001*
	PIGD	6 (12.5%)	16 (59.3%)		
	Indeterminate subtype	5 (10.4%)	4 (14.8%)		
Parkinson Asymmetry^		9 (18.75%)	2 (7.40%)	-5.21	< 0.001*
Symmetry index*^* *(Asymmetrical subjects)*		34 (70.4%)	7 (25.9%)	14.062	< 0.001*
Lower parkinsonism*^*		6 (12.5%)	11 (40.7%)	7.862	0.005*
MDS-UPDRS total score OFF		80.50 (61)	79 (21)	-0.574	0.566
MDS-UPDRS total score ON		61 (42)	71(21)	-1.512	0.130
MDS-UPDRS part I		16.5 (10)	16 (5)	-0.746	0.455
MDS-UPDRS Part II		16 (17)	18(8)	-0.519	0.604
MDS-UPDRS Part III –OFF		51(31)	45 (14)	-1.016	0.310
MDS-UPDRS Part III –ON		27 (21)	39 (10)	-3.038	0.002*
Rigidity OFF		8 (6)	8 (3)	-0.577	0.564
Rigidity ON		4.50 (5)	7(3)	-3.825	< 0.001*
Bradykinesia OFF		17 (16)	16 (6)	-0.017	0.987
Bradykinesia ON		8.50 (10)	14 (7)	-2.113	0.035*
PIGD OFF		6.50 (10)	9 (2)	-1.881	0.060
PIGD ON		4 (7)	9 (2)	-3.543	< 0.001*
Axial OFF		12.50 (12)	16 (4)	-2.195	0.028*
Axial Score ON		7 (9)	15 (4)	-5.066	< 0.001*
Tremor score OFF		14 (11)	5 (13)	-4.418	< 0.001*
Tremor score ON		9 (7)	5 (8)	-3.310	< 0.001*
Constancy of rest tremors off		2 (2)	0 (2)	-4.477	< 0.001*
Constancy of rest tremors on		1 (1)	0 (2)	-2.709	0.007*
H&Y OFF		2.5 (1.5-4)	3 (2–5)	-3.373	< 0.001*
H&Y ON		2 (1–3)	3 (2–5)	-4.758	< 0.001*
S&E ADL OFF		80 (17.50)	60 (10)	-4.208	< 0.001*
S&E ADL ON		90 (10)	60 (20)	-5.254	< 0.001*
Motor complication total score		5 (8)	4 (2)	-1.272	0.203
NFOG-Q OFF		26 (54.2%)	5 (18.5%)	9.056	0.003*
NFOG-Q ON		21(43.8%)	5 (18.5%)	4.857	0.028*
BBS OFF		47.50 (14)	33 (9)	-4.541	< 0.001*
BBS ON		54 (9)	36 (12)	-5.505	< 0.001*
TUG OFF		12.90 (12.25)	16.12 (9.74)	-1.761	0.078
TUG ON		10.65 (6.16)	13.55 (8.31)	-2.936	0.003*
10-MWT, Comfortable speed OFF (meter/sec)		0.797 (0.435)	0.712 (0.347)	-1.496	0.135
10-MWT, Comfortable speed ON (meter/sec)		0.91 (0.412)	0.83 (0.389)	-1.887	0.059
Urine incontinence scale		1 (3)	2 (1)	2.463	0.014*
IPAQ total score		1790 (1283.3)	417 (954)	5.603	< 0.001*
LEDD Mean (SD)**		631.48 (398.76)	415.74 (126.94)	3.45	< 0.001*
Dopa responsive (%)*^*		44 (91.7%)	7 (25.9%)	34.321	< 0.001*

AOO, age of onset; DOI, duration of illness; LEDD, Levodopa equivalent daily dose; MDS-UPDRS, Movement Disorder Society—Unified Parkinson’s Disease Rating Scale, PIGD postural instability –gait difficulty; H & Y, Hoehn and Yahr scale for Parkinson; S&E, Schwab and England ADL Scale; ADL, activities of daily living; NFOG-Q; new freezing of gait questionnaire; BBS, berg balance scale; TUG, time up and go test; 10 MWT, 10-meter walk test. IPAQ, international physical activity questionnaire,

^ Chi-square test is used

** T-test is used

*p value is significant

### Non-motor symptoms, quality of life and cognitive functions of PD and VaP

Compared to controls, patients with PD and VaP had significantly worse BDI, total NMSS and subscores of NMSS, except for perceptual problems/hallucinations of VaP group. Compared to VaP group, PD group had significantly worse NMSS total score (*p* = 0.030), sleep/fatigue (*p* = 0.031), mood/cognition (*p* = 0.006), and miscellaneous domains (*p* < 0.001), while VaP patients had significantly worse urinary domain than PD patients (*p* = 0.037). There were no significant differences in other domains of NMSS and BDI between PD and VaP groups. 33 (68.7%) patients with PD and 21 (77.8%) patients with VaP had depression with comparable frequency and severity types (*p* = 0.302).

Compared to controls, patients with PD and VaP showed significantly worse total PDQ-39 and its domains (*p* < 0.001), except for bodily discomfort for VaP group. Compared to patients with VaP, patients with PD had significantly worse total PDQ-39 (*p* = 0.047), emotional wellbeing (*p* = 0.044), stigma (*p* < 0.001), social support (*p* < 0.001) and bodily discomfort (*p* = 0.003), with no significant difference in other domains (Table 3).

**Table 3:** Non-motor functions and quality of life among people with Parkinson’s disease and vascular parkinsonism

	Parkinson’s disease (No. =48)	Vascular parkinsonism (No.=27)	Control (No. =29)	Kruskal-Wallis Test	Parkinson’s disease vs. Vascular parkinsonism^a^	Parkinson’s disease vs. control^a^	Vascular 541 vs. control^a^
	Median (Range/ IQR)	Median (Range/ IQR)	Median (Range/ IQR)	***P***	***P***	***P***	***p***
**Non-motor Symptoms Sclae**							
**NMSS total score**	55 (4-205/ 47)	39 (10–100/ 30)	7 (1–33/ 6)	< 0.001*	0.030*	< 0.001*	< 0.001*
Cardiovascular	1 (2)	2(3)	0(0)	< 0.001*	0.688	< 0.001*	< 0.001*
Sleep/fatigue	8 (8)	6 (6)	0 (1)	< 0.001*	0.031*	< 0.001*	< 0.001*
Mood/Cognition	12 (16)	6 (4)	3 (2)	< 0.001*	0.006*	< 0.001*	< 0.001*
Perceptual problems/ hallucinations	0 (1)	0 (0)	0 (0)	0.102			
Attention/memory	6 (10)	6 (9)	1 (1)	< 0.001*	0.820	< 0.001*	< 0.001*
Gastrointestinal tract	5 (8)	4 (8)	0 (1)	< 0.001*	0.319	< 0.001*	< 0.001*
Urinary	5.50 (11)	12 (6)	0 (1)	< 0.001*	0.037*	< 0.001*	< 0.001*
Sexual functions	2 (4)	2 (0)	0 (0)	< 0.001*	0.652	< 0.001*	< 0.001*
Miscellaneous	5.50 (7)	1 (2)	0 (1)	< 0.001*	< 0.001*	< 0.001*	0.003*
**BDI total score**	17 (0–39)	16.00 (5–33)	4.50 (2–9)	< 0.001*	0.675	< 0.001*	< 0.001*
**PDQ-39**							
PDQ total score^	38.72 (30.10)	31.25 (14.95)	7.60 (3.56)	< 0.001*	0.047*	< 0.001*	< 0.001*
Mobility	42.50 (36.88)	60.00 (35.00)	2.50 (3.75)	< 0.001*	0.055	< 0.001*	< 0.001*
ADL	50.00 (53.12)	45.83 (29.17)	0.00 (0.00)	< 0.001*	0.103	< 0.001*	< 0.001*
Emotional wellbeing	39.58 (37.50)	29.17 (20.83)	20.83 (8.33)	< 0.001*	0.044*	< 0.001*	< 0.001*
Stigma	68.75 (48.44)	31.25 (50.00)	0.00 (0.00)	< 0.001*	< 0.001*	< 0.001*	< 0.001*
Social support	16.66 (37.50)	0.00 (8.33)	0.00 (0.00)	< 0.001*	< 0.001*	< 0.001*	0.002*
Cognition	25.00 (31.25)	37.50 (18.75)	12.50 (18.75)	< 0.001*	0.266	< 0.001*	< 0.001*
Communication	33.33 (39.59)	25.00 (25.07)	0.00 (0.00)	< 0.001*	0.388	< 0.001*	< 0.001*
Bodily discomfort	41.66 (25.00)	33.33 (16.67)	25.00 (16.73)	< 0.001*	0.003*	< 0.001*	0.651
**Cognitive Scales**							
MOCA total score	22(6–29)	19.00 (12–24)	24 (17–27)	< 0.001*	0.015*	0.014*	< 0.001*
FAB total score	13 (2–17)	9.00 (6–14)	13.50 (9–17)	< 0.001*	0.001*	0.091	< 0.001*
WMS total score	41.75 (11.4)	38 (13.0)	47 (10.3)	< 0.001*	0.006*	0.040*	< 0.001*
Verbal fluency of ACE-III	1 (3)	0 (0)	1 (1)	< 0.001*	< 0.001*	0.974	< 0.001*
Clock drawing of ACE-III	3 (3)	3 (2)	5(2)	< 0.001*	0.602	< 0.001*	< 0.001*

NMSS: non-motor symptoms scale; PDQ-39: Parkinson’s disease questionnaire-39, ADL: *activities of daily living*, MOCA: Montreal cognitive assessment; FAB: Frontal Assessment Battery; BDI: Beck depression inventory; WMS: Wechsler memory scale; ACE-III: Addenbrooke’s Cognitive Examination -III

^a^ Mann Whitney U test was used

*p-value is significant

PD and VaP groups were significantly worse on the MoCA, WMS and clock drawing test than controls. Only VaP group had significantly worse FAB and verbal fluency than controls (*p* < 0.001). Compared to PD group, VaP group had significantly worse MOCA (*p* = 0.015), FAB (*p* < 0.001), verbal fluency (*p* < 0.001) and WMS (*p* = 0.006) scores and similar clock drawing test scores (Table 3).

### Acute versus insidious VaP

The insidious group had significantly worse rigidity OFF, PIGD OFF (*p* = 0.02), total NMSS (*p* = 0.02), cardiovascular (*p* = 0.03), gastrointestinal (*p* = 0.02), and sexual functions (*p* = 0.03) domains of the NMSS, BDI (*p* = 0.02), and stigma (*p* = 0.04) and better body discomfort (*p* = 0.04). Both types showed similar MRI vascular and atrophy scores (Supplementary Table 1).

### Laboratory differences between PD and VaP

VaP group showed significantly lower serum haemoglobin (*p* = 0.005), albumin (*p* < 0.001), and HbA1c (*p* = 0.014) and higher uric acid (*p* = 0.015), cholesterol (*p* = 0.002) and low-density lipoprotein (LDL) (*p* = 0.012) than PD group (Supplementary Table 2).

### Neuroimaging differences between PD and VaP

**Fig. 1:**
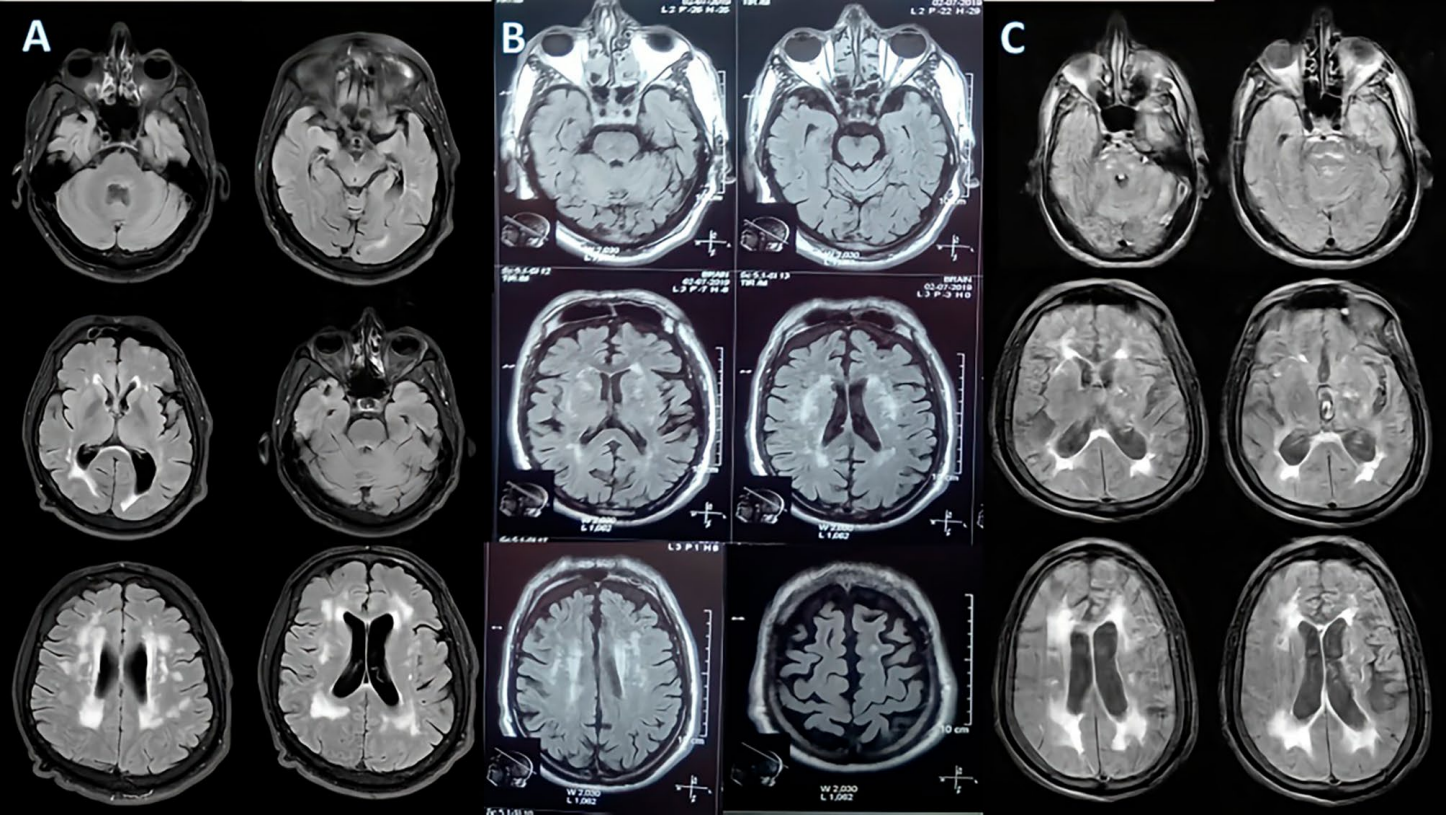
Examples of three patterns of white matter hyperintensities demonstrated in Brain MRI FLAIR: **(A)** bilateral cerebral periventricular bright signal foci and confluent patches, **(B)** bilateral cerebral periventricular and basal ganglia signal abnormalities, **(C)** bilateral cerebral periventricular, basal ganglia and pontine hyperintensities

The VaP group had a significantly higher Fazekas scale (*p* < 0.001), Scheltens’ scale (*p* < 0.001) and visual rating scale for atrophy (*p* < 0.001) than the PD group, implying more severe white matter ischemic changes and brain atrophy (Supplementary Table 3). Seventeen patients with VaP (62.96%) had Fazekas grade 2, eight patients (29.63%) had grade 3, two patients (7.4%) had grade 1, and no patient had grade 0, while 18 patients with PD (37.5%) had Fazekas grade 0, 24 patients (50%) had grade 1, 6 patients (12.5%) had grade 2, and no patient had grade 3. Three patterns of brain white matter hyperintensities were identified including: bilateral cerebral periventricular hyperintense foci and confluent patches (7 patients, 25.9%), bilateral cerebral periventricular and basal ganglia hyperintensities (8 patients, 29.6%), and bilateral cerebral periventricular, basal ganglia and pontine hyperintensities (12 patients, 44.4%) (Fig. 1). 20 patients with VaP (74.07%) had basal ganglionic ischemic changes, while only 3 patients with PD (6.25%) had these changes.

TCCD could be performed for 47 patients with PD, 24 patients with VaP, and all controls, while 3 patients with VaP and one patient with PD had poor transtemporal window. The VaP group had significantly higher IMT of the CCA than the PD and control groups (*p* = 0.011 and < 0.001, respectively) but similar carotid plaques. The VaP group had a significantly lower PSV than the controls but was comparable to the PD group. The average and PCA MFV were significantly lower in the VaP group than in the PD and control groups. Additionally, the VaP group had a significantly higher PI of the MCA than the PD group (*p* = 0.031) and controls (*p* = 0.004) and a higher PI of the PCA than controls (Table 4). Compared to controls, the PD group had significantly impaired BHI of both sides MCA (*p* = 0.012 and *p* < 0.001) and PCA (*p* < 0.001 and *p* = 0.004), and the VaP group had impaired BHI of both PCA (*p* < 0.001 and *p* = 0.019). However, there were no significant differences between the PD and VaP groups regarding BHI. Hyperechogenicity of the SN was more significantly detected in PD group (43 patients (91.5%)) than in VaP group (5 patients (20.8%)) (*p* < 0.001) and controls (3 (10.3%)) (*p* < 0.001) (Table 4).

**Table 4:** Carotid duplex and transcranial colour-coded doppler findings among all study groups

	Parkinson Disease	Vascular Parkinsonism	Control	Kruskal-Wallis Test	PD vs. VaP	PD vs. control	VaP vs. control
	Median (Range)	Median (Range)	Median (Range)	***P***	***P***	***P***	***p***
**Carotid Duplex**							
**Number**	47	24	29				
Right CCA IMT	0.91 (0.30)	1.10 (0.56)	0.80 (0.20)	< 0.001*	0.011*	0.004*	< 0.001*
Left CCA IMT	1 (0.35)	1.15 (0.40)	0.8 (0.20)	< 0.001*	0.011*	< 0.001*	< 0.001*
Right ICA PSV	33.20 (15.7)	29.35 (12.8)	41.70 (15.8)	0.012*	0.253	0.022*	0.006*
Left ICA PSV	34.40 (18.8)	28.10 (9.40)	39.90 (16.9)	0.004*	0.049*	0.054	0.001*
Carotid Plaques	5 (10.4%)	4 (14.8%)	0 (0.0%)	0.120			
**Transcranial colour-coded doppler**							
Number (poor widow)	47 (1)	24 (3)	29 (0)				
**Mean Flow Velocity (MFV)**							
Average MFV	52.64 (72.45)	45.95 (88.83)	59.30 (28.65)	0.002*	0.038*	0.019*	0.002*
Right MCA MFV	60.80 (22.1)	59.60 (29.2)	63.10 (11.60)	0.373			
Left MCA MFV	68.60 (29.3)	56.90 (24.7)	67 (25.6)	0.066			
Right PCA MFV	40.90 (11.7)	41.45 (15.0)	47.70 (9.4)	0.002*	0.666	< 0.001*	0.035*
Left PCA MFV	42 (16.6)	34.30 (16.3)	50.20 (18.9)	< 0.001*	0.038*	0.004*	< 0.001*
**Pulsatility Index**							
Right MCA pulsatility index	0.80 (0.36)	0.99 (0.43)	0.77 (0.19)	0.016*	0.031*	0.410	0.004*
Left MCA pulsatility Index	0.86 (0.35)	0.80 (0.45)	0.74 (0.23)	0.152			
Right PCA pulsatility index	0.75 (0.34)	0.89 (0.42)	0.69 (0.26)	0.012*	0.076	0.094	0.004*
Left PCA pulsatility Index	0.84 (0.40)	0.87 (0.46)	0.71 (0.16)	0.012*	0.511	0.010*	0.011*
**Breath Holding Index (BHI)**							
Right MCA BHI	-0.86 (1.39)	-0.60 (0.99)	-0.14 (0.69)	0.036*	0.261	0.012*	0.195
Left MCA BHI	-0.89 (1.07)	-0.65 (1.25)	-0.13 (0.72)	< 0.001*	0.084	< 0.001*	0.110
Right PCA BHI	-0.80 (0.99)	-0.72 (1.36)	-0.02 (0.32)	< 0.001*	0.947	< 0.001*	< 0.001*
Left PCA BHI	-0.65 (1.96)	-0.39 (0.63)	-0.15(1.26)	0.007*	0.282	0.004*	0.019*
**Substantia nigra hyperechogenicity**	43 (91.5%)	5 (20.8%)	3 (10.3%)	< 0.001*	< 0.001*	< 0.001*	0.288

CCA: common carotid artery; IMT: intima-media thickness; PSV: peak systolic velocity; ICA: internal carotid artery; MCA: middle cerebral artery; MVF: mean flow velocity; BH: breath holding; PCA: posterior cerebral artery; BHI: breath holding index, VaP: vascular parkinsonism

^chi test is used

*p-value is significant

### Correlations of motor, non-motor symptoms and quality of life in PD and VaP

Among VaP group, MDS-UPDRS-III -Off was significantly correlated with age, duration, and number of vascular risk factors (*r* = 0.541, *p* = 0.006). MoCA was correlated with FAB and depression. Total NMSS was significantly correlated with age, BDI, MDS-UPDR-III Off, and FAB. The PDQ scores were significantly correlated with the MDS-UPDRS-Off, H&Y Off, NMSS and BDI scores (*r* = 0.429, *p* = 0.025; *r* = 0.477, *p* = 0.012; *r* = 0.621, *p* = 0.001; and *r* = 0.529, *p* = 0.005, respectively) (Table 5). Average MFV was correlated with PIGD Off and FAB scores (*r* = 0.473, *p* = 0.013, and *r* = -0.390, *p* = 0.045, respectively).

**Table 5:** Correlations of clinical characteristics and quality of life of Parkinson’s disease and vascular parkinsonism

		MDS -UPDRS III Off	MoCA	NMSS	PDQ
Vascular Parkinsonism					
Age	*Spearman*	0.417	-0.159	0.412	0.141
	*P-value*	0.031	0.427	0.033	0.482
AOO	*Spearman*	0.366	-0.181	0.311	0.061
	*P-value*	0.061	0.365	0.114	0.764
DOI	*Spearman*	0.406	-0.082	0.362	0.179
	*P-value*	0.036	0.684	0.064	0.372
BDI	*Spearman*	0.362	-0.382	0.791	0.529
	*P-value*	0.064	0.049	< 0.001	0.005
MDS-UPDRS III OFF	*Spearman*		-0.252	0.442	0.429
	*P-value*		0.205	0.021	0.025
H&Y OFF	*Spearman*	0.136	-0.314	0.357	0.477
	*P-value*	0.499	0.111	0.067	0.012
FAB	*Spearman*	-0.261	0.659	-0.397	-0.328
	*P-value*	0.189	< 0.001	0.041	0.095
Number of vascular risk factors	*Spearman*	0.514	-0.360	0.225	0.232
	*P-value*	0.006	0.065	0.259	0.245
Fazekas total score	*Spearman*	-0.040	0.136	0.088	0.054
	*P-value*	0.842	0.500	0.662	0.787
Schelten total score	*Spearman*	-0.143	0.007	-0.161	0.057
	*P-value*	0.477	0.973	0.424	0.776
**Parkinson’s disease**					
Age	*Spearman*	-0.031	-0.134	0.060	-0.071
	*P-value*	0.836	0.362	0.683	0.634
AOO	*Spearman*	-0.203	-0.02	-0.041	-0.259
	*P-value*	0.166	0.893	0.780	0.075
DOI	*Spearman*	0.470	-0.063	0.320	0.472
	*P-value*	< 0.001	0.675	0.028	0.001
BDI	*Spearman*	0.587	-0.334	0.673	0.723
	*P-value*	< 0.001	0.020	< 0.001	< 0.001
MDS-UPDRS III OFF	*Spearman*		-0.318	0.604	0.778
	*P-value*		0.028	< 0.001	< 0.001
H&Y OFF	*Spearman*	0.843	-0.33	0.433	0.688
	*P-value*	< 0.001	0.022	0.002	< 0.001
FAB	*Spearman*	-0.353	0.7	-0.243	-0.309
	*P-value*	0.015	< 0.001	0.099	0.034
Number of vascular risk factors	*Spearman*	-0.016	0.041	0.274	0.164
	*P-value*	0.914	0.78	0.059	0.265
Fazekas total score	*Spearman*	0.007	-0.113	0.085	-0.158
	*P-value*	0.962	0.445	0.566	0.283
Schelten total score	*Spearman*	-0.099	-0.028	0.083	-0.169
	*P-value*	0.502	0.852	0.575	0.250

AOO: age of onset; DOI: duration of illness; BDI: Beck depression inventory; MDS-UPDRS: movement disorder society – unified Parkinson’s disease rating scale; H&Y: Hoehn, and Yahr scale; NMSS: non-motor symptoms scale; PIGD = postural instability and gait disorder; FAB: Frontal Assessment Battery; MOCA: Montreal cognitive assessment; SN: Substantia Nigra, WMS: Wechsler memory scale; PDQ-39: Parkinson’s disease questionnaire-39. BHI: breath holding index

After Bonferroni correction, the *p*-value is significant if < 0.004

Among PD group, MDS-UPDRS-III Off was significantly correlated with duration, H&Y Off, BDI (*p* < 0.001) and FAB (*p* = 0.015). MoCA scores were significantly correlated with BDI, MDS-UPDRS-III Off, H&Y Off, and FAB scores. Total NMSS was significantly correlated with duration, BDI, MDS-UPDRS III Off and H&Y Off. The PDQ was significantly correlated with duration (*p* = 0.001), BDI, MDS-UPDRS-III Off, H&Y Off (*p* < 0.001), total NMSS (*r* = 0.790, *p* > 0.001) and FAB scores (*p* = 0.034). Correlations with *p* < 0.004 are significant after Bonferroni correction (Table 5).

## Discussion

The current study comprehensively characterized the clinical and radiological differences between VaP and PD. In addition to confirming the motor and non-motor characteristics of VaP that differentiate it from PD, the study described its motor subtypes, impaired QoL and factors associated with motor, NMSs and impaired QoL. Moreover, it demonstrated the cerebral hemodynamic changes associated with VaP. This study included matched ages, gender and education years for proper comparison and to avoid previous studies’ limitations [4]. 

The present study demonstrated that VaP was associated with more vascular risk factors, similar to previous studies [4, 37–39]. Moreover, vascular risk factors were correlated with motor severity. Furthermore, laboratory tests showed higher vascular risk factors, such as serum uric acid and LDL, among patients with VaP. Similarly, one study showed higher serum uric acid in VaP than in PD, which could also be explained by the association between low serum uric acid and developing PD [40]. 

On the other hand, family history and consanguinity were significantly more frequent among PD patients, in contrast to a previous study that showed no significant difference, [4] implying the acquired nature of VaP. Most of the studies reported more male predominance and older age among VaP than PD [37, 38]. This may be explained by the higher stroke incidence in males and the protective role of estrogen among females [41]. Moreover, it confirmed the older age of onset and shorter duration of VaP compared to age-matched PD, similar to previous studies of matched [42] and unmatched ages [37–39]. 

The characteristic motor features of VaP have been confirmed, including lower body predominance, more symmetrical symptoms, worse motor scores, wide-based gait, worse balance, more postural instability, less tremor, less satisfactory response to levodopa and associated pyramidal signs, in agreement with previous studies. Moreover, the PIGD type is more frequent among VaP (about 60%), but TD might also be present in 25.9%, denoting the overlap with PD. TD with VaP could be related to the prevalence of postural upper limbs and jaw tremor [37]. 

Significant differences between VaP and PD were more pronounced during the On state, implying different responses to levodopa [4, 37, 39, 43, 44]. Additionally, the current study showed worse balance and physical activity among VaP. Off scores of total and motor MDS-UPDRS, bradykinesia and rigidity were comparable in both groups in contrast to other studies, which could be explained by the lower severity, duration, and age of PD group in this study. Meanwhile, other studies found no significant difference in rigidity and bradykinesia between PD and VaP [37, 44]. Consequently, motor features such as tremor, balance disturbance, axial symptoms and PIGD are more consistent differentiating features between VaP and PD in On and Off states.

Freezing of gait is one of the common features of VaP. However, this study showed higher gait freezing in PD group than in VaP group, in contrast to previous studies [7, 37, 39]. Meanwhile, other studies reported no difference in gait dysfunction and freezing between VaP and PD [44, 45]. This discrepancy could be attributed to the greater cognitive impairment among patients with VaP that might affect the recognition and reporting of freezing and the short duration of illness of VaP in our study. Additionally, gait freezing is more closely related to the localization of lesions that might differ among groups [46, 47]. 

Cognitive impairment was more prominent in the VaP of different domains, especially frontal lobe dysfunction, with less impairment of visuospatial functions than PD and healthy individuals, while patients with PD showed preserved frontal functions. Similarly, previous studies reported more global cognitive impairment in VaP [7, 38, 48]. Benítez-Rivero et al. described the same findings after adjustment for age, in addition to a greater effect on visuospatial functions of patients with PD [42]. However, the current study found more impaired memory among patients with VaP in contrast to previous studies that showed comparable memory tasks [42]. This could be attributed to ischemia-related changes in the functional connectivity of the caudate nucleus with the cingulate cortex, inducing severe executive/frontal lobe dysfunction [49, 50]. 

Few studies have described the non-motor aspects of VaP. VaP had worse total and domains of NMSS and urinary symptoms but better sleep/fatigue, mood/cognition, and miscellaneous domains than PD. Benítez-Rivero et al. reported less frequent NMSs in the VaP than in the PD but non-significantly worse NMSs in the VaP than in the controls [42]. On the other hand, Raimundo et al. reported a non-significantly higher prevalence of NMSs, particularly sleep/fatigue and mood/cognition, in VaP than in PD, but VaP patients were older and of small number [51]. Additionally, the PRIAMO study reported a high prevalence of different NMSs in the VaP among patients with PD and other atypical parkinsonism but with unmatched ages [52]. Urinary dysfunction and urinary incontinence are characteristic features of VaP in concordance with previous studies [4, 38, 39, 43, 48]. Remarkably, total NMSS was related to motor severity, age and frontal cognitive dysfunction but not disease duration or stage such as PD, implying different underlying pathogenesis. In contrast to NMSS, the total score of MDS-UPDRS part I did not show a significant difference between both groups. This could be explained by the different structures and contents of both instruments and the variable association between them according to the severity of NMSs [53]. 

Patients with VaP showed worse QoL than normal individuals and better total and domains of PDQ-39 than PD patients, except for mobility and cognition, which was related to motor severity, NMS and depression similar to PD patients, but not to duration [23]. Similarly, the PRIAMO study showed impaired QoL in VaP that was also related to disease severity and motor scores [52]. Consequently, management of QoL determinants is essential for better care of patients with VaP. Interestingly, patients with VaP had better stigma and social support domains than patients with PD.

Vascular lesions in neuroimaging are essential for diagnosing VaP. Previous studies confirmed more vascular lesions and atrophy in neuroimaging in VaP than in PD, but few studies used visual rating scales for WMLs and correlated abnormalities with clinical characteristics [4, 37–39, 42]. Fazekas scores were higher in VaP than in PD, in accordance with other studies [4]. We also used the Schelten scale with its regional parts, which confirmed higher WMLs in the VaP. The periventricular ischemic changes represent the most brain MRI changes in VaP, followed by deep white matter, basal ganglionic and, to a lesser extent, infratentorial lesions. Similar findings were reported by a clinicopathological study [44]. Demirkiran et al. reported that all patients with VaP had ischemic lesions, mainly in subcortical white matter and, to a lesser extent, basal ganglia and brainstem in brain MRI, while 70% of patients with PD had normal MRIs [37]. Rath and colleagues reported that periventricular ischemic change, generalized brain atrophy, and multiple lacunar infarcts were the most common radiological abnormalities found significantly more frequently in VaP [38]. The prevalence of ischemic brain MRI changes in PD must be considered to prevent incorrect diagnosis of VaP [54]. 

However, there was no significant correlation between WMLs and motor severity. In contrast, Chen et al. reported a significant correlation with motor severity, daily activity and UPDRS-I, suggesting that disruption of cortical subcortical circuits by WMLs is the underlying cause of these symptoms [55]. In the current study, patients with VaP had a younger age and shorter duration, which may explain this variability in addition to the small number of patients in different studies. Moreover, using more sensitive neuroimaging techniques and rating scales for white matter is required to investigate this association.

Transcranial sonography has been suggested as a supplementary tool to differentiate VaP from PD [56]. TCCD has evident advantages, including non-invasiveness, speed of examination, and widespread availability [56]. Moreover, hyperechogenicity of the SN is a sensitive tool in the differentiation between PD and other parkinsonian syndromes [57]. Our study showed that patient with PD had significantly higher SN hyperechogenicity than patients with VaP and controls. SN hyperechogenicity was detected in only 20.8% of patients with VaP and in 90.1% of patients with PD, similar to the results of a previous study [58]. Another study reported its presence in 42% of patients with VaP [59]. Therefore, SN hyperechogenicity can be used as a simple cost-effective method to differentiate between VaP and PD.

Detecting cerebral hemodynamic vascular changes is another tool to differentiate VaP from PD and understand the underlying pathogenesis. Remarkably, low flow velocity, high PI and impaired BHI were detected in the VaP, especially the PCA, implying increased cerebral vascular resistance and decreased vasoreactivity distally as a marker of small vessel disease. Similarly, Tsai et al. reported a higher PI with VaP than with PD and controls but with similar intracranial flow velocities [58]. Another study reported a higher PI with VaP than with PD, supporting its use to confirm the diagnosis of VaP [60]. Remarkably, average flow velocity showed a correlation with motor (PIGD Off) and cognitive dysfunction (FAB). On the other hand, lower intracranial flow velocity has been detected in PD patients than in controls [61, 62]. 

There was no significant difference between the PD and VaP groups regarding BHI, while both the PD and VaP groups had significantly impaired BHI compared with the control group, suggesting the presence of impaired vasomotor reactivity in both groups. Previous studies reported impaired BHI and cerebrovascular reactivity compared with controls [63]. The impairment of vasomotor reactivity in VaP may be due to associated vessel wall disease. These associated vascular changes may disrupt the basal ganglia-thalamocortical circuits, resulting in the clinical features of VaP [58]. 

The small number of participants among different groups is one of the study limitations, despite including larger numbers than other previous studies. Expectedly, the patients with VaP were older, but this was not statistically difference. Specific NMSs were not included such as olfactory dysfunction and rapid eye movement sleep behavior disorder. Additional limitations include the presence of a poor transtemporal window for TCCD among some patients and the lack of magnetic resonance angiography. The use of more advanced functional neuroimaging and DAT scan is required for assessing patients with PD and VaP, which were not available in our country. The strengths of this study include comprehensive clinical and neuroimaging characteristics of VaP compared to age- and gender-matched PD and controls, identifying the correlations of motor, nonmotor and QoL in VaP, comprehensive assessment of cerebral vascular hemodynamics and the use of visual rating scales of WML.

The diagnosis of insidious VaP remains challenging and debatable due to overlapping features with other diseases e.g., NPH and higher-level gait disorders, lack of specific clinical features or diagnostic tests, inadequate diagnostic criteria, lack of pathologically confirmed angiopathy, and the possibility of underlying specific genetic syndromes [6, 64]. Additionally, several caveats were reported for the commonly used criteria by Zjilmans et al., particularly the poorly defined clinical and neuroimaging criteria and its reliance on a cohort of mixed pathology [6, 65]. 

Despite the reported cases of parkinsonism-related genetic leukoencephalopathy, these cases represent a small percentage of patients with cerebral small vessel disease, with overlapped clinical features and neuroimaging [66, 67], indicating the need for genetic testing of patients’ cohorts to confirm its frequency among patients diagnosed currently with VaP. Most cerebral small vessel disease cases are attributed to interaction between environmental factors and multiple genetic variants, while monogenic variants represent a minor percentage (up to 5%) [67, 68]. Furthermore, arteriopathy might have a variable role in these genetic leukoencephalopathies, ranging from a primary role to minor or no causal evidence [66]. 

Therefore, VaP is considered a heterogenous syndrome, with different underlying pathogeneses, and with no or minor role of vascular changes, implying the need for reconstructing this syndrome, proper dissection from other diagnoses or underlying genetic leukoencephalopathies and using different description instead of VaP [6]. “Adult leukoencephalopathy-associated parkinsonism” might be suggested for those patients, if clinical criteria of parkinsonism exist (e.g., bradykinesia plus rigidity or tremor), that might involve heterogeneous conditions. Until resolving these challenges, further comprehensive studies might help identify this clinical syndrome using the existing criteria as a starting point with considering its caveats or identifying new criteria [64, 67]. 

## Conclusions

The current study confirmed the clinical motor, non-motor, brain MRI and transcranial sonography characteristics of VaP that might differentiate it from PD. It also identified the impaired QoL in VaP that was correlated with motor and nonmotor features. Addressing and managing motor and NMSs are essential for better QoL and care of patients with VaP. Furthermore, this study demonstrated impaired distal cerebral vascular changes in VaP, which were correlated with motor and cognitive dysfunction, suggesting a role of vascular dysfunction in its pathogenesis. However, there was a lack of correlation of WMLs with disease characteristics, implying the need for further studies to explore the role of vascular lesions and to reconstruct properly this clinical syndrome.

### Electronic supplementary material

Below is the link to the electronic supplementary material.

Supplementary Material 1

## Data Availability

The data and materials used along the current study are available from the corresponding author on reasonable request.

## Abbreviations

ACE-III: Addenbrooke’s cognitive examination III
BBS: Berg Balance Scale
BDI: Beck Depression Inventory
BHI: Breath holding index
CCA: Common carotid artery
CVR: Cerebral vasomotor reactivity
H & Y: Hoehn and Yahr
ICIQ-SF: International Consultation on Incontinence Questionnaire-Short Form
IMT: Intimal medial thickness
LEDD: Levodopa equivalent daily dose
LDL: Low-denisty lipoprotein
MCA: Middle cerebral artery
MDS-UPDRS: Movement disorders society – unified Parkinson’s disease rating scale
MDS: Movement disorders society
MFV: Mean flow velocity
MoCA: Montreal cognitive assessment
MRI: Magnetic resonance imaging
NFOG-Q: New freezing of gait questionnaire
NMSS: Non motor symptoms scale
NPH: Normal pressure hydrocephalus
PCA: Posterior cerebral artery
PD: Parkinson’s Disease
PI: Pulsatility index
PIGD: Postural instability and Gait disorder
PSV: Peak systolic velocity
QoL: Quality of life
S&E-ADL: Schwab and England activities of daily living scale
SN: Substantia nigra
TCCD: Transcranial color-coded Doppler
TUG: time up and go
VaP: Vascular Parkinsonism
WML: White matter lesion
WMS: Wechsler memory scale
