# Supplementary material for: The clinical and neuroimaging differences between vascular parkinsonism and Parkinson’s disease: a case-control study
Source: BMC Neurol. 2024 Feb 6;24:56. doi: 10.1186/s12883-024-03556-9 (PMC10845551; doi:10.1186/s12883-024-03556-9)
Supplement: Supplementary file 1 — Supplementary Material 1 [file 12883_2024_3556_MOESM1_ESM.docx]

**Supplementary tables**

**Supplementary Table 1: Differences between acute and insidious vascular parkinsonism**

|  | Acute Vascular Parkinsonism  (Number 10) | Insidious Vascular Parkinsonism  (Number 17) | Mann Whitney test | |
| --- | --- | --- | --- | --- |
|  | Median (IQR) | Median (IQR) | Z | p |
| Age | 68 (18.75) | 65 (15.5) | -0.58 | 0.56 |
| Educational years | 7.5 (11.25) | 8 (9) | -0.03 | 0.98 |
| AOO | 65 (18) | 64 (11) | 0.00 | 1.00 |
| DOI | 2 (1.5) | 4 (3.25) | -1.42 | 0.15 |
| LEDD | 425 (0) | 425 (100) | -1.13 | 0.26 |
| MDS-UPDRS total score OFF | 75.5 (29) | 86 (19.5) | -1.28 | 0.20 |
| MDS-UPDRS total score ON | 72 (26) | 71 (24.5) | -0.43 | 0.67 |
| MDS-UPDRS Part II | 15 (8.75) | 16 (4.5) | -1.45 | 0.15 |
| MDS-UPDRS Part III -OFF | 43 (8.5) | 49(14.5) | -1.16 | 0.25 |
| MDS-UPDRS Part III -ON | 39.5 (9) | 38 (13) | -0.68 | 0.50 |
| Rigidity OFF | 8 (1.25) | 10 (3.5) | -2.334 | 0.02* |
| Rigidity ON | 7 (2) | 8 (4) | -1.045 | .296 |
| Bradykinesia OFF | 18 (7.25) | 16 (6) | -0.66 | 0.51 |
| Bradykinesia ON | 14.5 (5) | 12 (7.5) | -1.14 | 0.26 |
| PIGD OFF total score | 9 (1.25) | 10 (4) | -2.36 | 0.02* |
| PIGD ON total score | 9 (1.25) | 9 (5) | -0.56 | 0.57 |
| Axial Score OFF | 16 (3.5) | 16 (6.5) | -0.81 | 0.42 |
| Axial Score ON | 16 (4) | 15 (7.5) | -0.05 | 0.96 |
| Tremor score ON | 2.5 (9.25) | 7 (13) | -0.58 | 0.56 |
| DYSKINESIAS | 0 (0) | 0 (0) | 0.00 | 1.00 |
| H&Y OFF | 3 (1.25) | 3 (1) | -0.71 | 0.48 |
| H&Y ON | 2.75 (1.25) | 3 (1.5) | -0.29 | 0.78 |
| Schwab and England ADL OFF | 60 (35) | 55 (17.5) | -0.75 | 0.45 |
| Schwab and England ADL ON | 65 (35) | 60 (17.5) | -0.61 | 0.54 |
| Motor complication total score | 3 (1) | 4 (2) | -1.40 | 0.16 |
| NFOG_OFF | 0 (0.0%) | 5 (29.4%) | 3.610 | .057 |
| NFOG_ON | 0 (0.0%) | 5 (29.4%) | 3.610 | .057 |
| BBS OFF | 34.5 (14.5) | 32 (13.5) | -1.48 | 0.14 |
| BBS ON | 39.5 (11) | 35 (18) | -1.31 | 0.19 |
| TUG OFF | 16.28 (5.25) | 16 (13.9) | -0.20 | 0.84 |
| TUG ON | 12.17 (4.0275) | 15.19 (15.345) | -0.30 | 0.76 |
| (10-MWT) Comfortable speed OFF (meter/sec) | 0.85 (0.27) | 0.83 (0.46) | -0.88 | 0.38 |
| (10-MWT) Comfortable speed ON (meter/sec) | 1.14 (0.58) | 1 (0.73) | -0.98 | 0.33 |
| Total score of urine incontinence scale | 9 (8.25) | 9.5 (4.75) | -1.618 | 0.106 |
| IPAQ total score | 516 (949) | 417 (877.5) | -0.96 | 0.34 |
| **Non -motor symptoms scale** |  | | | |
| NMSS total score | 28.5 (25) | 47 (43.5) | -2.31 | 0.02* |
| Cardiovascular | 1 (2) | 4 (4) | -2.16 | 0.03* |
| Sleep/fatigue | 5.5 (7) | 6 (4) | -0.43 | 0.67 |
| Mood/Cognition | 6 (1) | 9 (10.5) | -1.87 | 0.06 |
| Perceptual problems/ hallucinations | 0 (1) | 0 (0) | -0.93 | 0.35 |
| Attention/memory | 5.5 (9) | 6 (9) | -0.03 | 0.98 |
| Gastrointestinal tract | 1 (5.25) | 6 (5.5) | -2.42 | 0.02* |
| Urinary | 9 (11.25) | 12 (5) | -1.74 | 0.08 |
| Sexual functions | 2 (1.25) | 2 (6) | -2.21 | 0.03* |
| Miscellaneous | 0.5 (1.25) | 1 (4) | -1.93 | 0.05 |
| **PDQ-39** |  | | | |
| PDQ total score | 31.45 (15.378) | 31.09 (13.34) | -0.65 | 0.51 |
| Mobility | 57.5 (50) | 60 (35) | -0.93 | 0.35 |
| ADL | 47.92 (40.63) | 40 (27.0835) | -0.05 | 0.96 |
| Emotional wellbeing | 25 (10.42) | 29.17 (20.83) | -1.25 | 0.21 |
| Stigma | 25 (31.25) | 43.75 (28.125) | -2.04 | 0.04* |
| Social support | 0 (4.17) | 0 (8.335) | -0.68 | 0.49 |
| Cognition | 37.5 (14.0625) | 31.25 (25) | -0.33 | 0.74 |
| Communication | 20.83 (18.75) | 25 (33.37) | -0.35 | 0.72 |
| Bodily discomfort | 33.33 (0) | 25 (20.87) | -2.03 | 0.04* |
| **Neuropsychological** |  | | | |
| MOCA total score | 18.5 (3.5) | 19 (5.5) | -0.38 | 0.70 |
| FAB total score | 9.5 (3.25) | 9 (3) | -0.48 | 0.63 |
| BDI total score | 14.5 (7.75) | 20 (9) | -2.34 | 0.02* |
| WMS total score | 37.75 (13.25) | 39.5 (13.5) | -0.40 | 0.69 |
| **MRI Brain** |  | | | |
| Fazekas scale |  | | | |
| Fazekas scale total | 2 (1.25) | 2(1) | -0.68 | 0.50 |
| Scheltens’ total score | 12 (7.5) | 12 (6) | -2.10 | 0.39 |
| Visual rating scale for Atrophy total score | 0(2) | 0(2) | -0.00 | 0.93 |
| **Laboratory investigations** |  | | | |
| HbA1c % | 5.75 (1.175) | 5.35 (0.75) | -0.23 | 0.82 |
| Uric acid (mg/dL) | 6 (2.675) | 7 (2) | -1.25 | 0.21 |
| Cholesterol (mg/dL) | 210 (93) | 186 (60) | -0.90 | 0.37 |
| Triglycerides (mg/dL) | 112 (58.75) | 99.5 (29) | -0.20 | 0.84 |
| LDL (mg/dL) | 118 (66.5) | 116.5 (65.1) | -0.60 | 0.55 |
| HDL (mg/dL) | 43 (11) | 39.45 (21.5) | -1.01 | 0.31 |
| **Transcranial color-coded duplex** |  | | | |
| Number of good window patients (poor window) | 8 (2) | 16 (1) |  |  |
| Right CCA IMT | 1.15 (0.575) | 1.1 (0.5975) | -0.03 | 0.98 |
| Left CCA IMT | 1.1 (0.325) | 1.2 (0.505) | -0.10 | 0.92 |
| Right CCA PSV | 29.15 (15.15) | 28.25 (9.2) | -0.08 | 0.94 |
| Left CCA PSV | 29.95 (8.775) | 29.5 (20.125) | -0.25 | 0.80 |
| Right ICA PSV | 31.1 (10.625) | 27.7 (14.55) | -0.73 | 0.47 |
| Left ICA PSV | 31 (11.125) | 27.1 (8.65) | -0.25 | 0.80 |
| Mean Flow Velocity (MFV) |  | | | |
| Right MCA MVF | 60.65 (25.925) | 59.6 (35.5) | -0.31 | 0.76 |
| Left MCA MVF | 48.15 (11.825) | 59.15 (26.775) | -1.23 | 0.22 |
| Right PCA MFV | 38.7 (11.15) | 42.6 (22.175) | -1.23 | 0.22 |
| Left PCA MFV | 32.1 (14.225) | 38.7 (24.2) | -1.69 | 0.09 |
| Pulsatility Index |  | | | |
| Right MCA Pulsatility Index | 0.925 (0.4975) | 1.025 (0.48) | -0.52 | 0.60 |
| Left MCA Pulsatility Index | 0.88 (0.3425) | 0.73 (0.54) | -0.25 | 0.81 |
| Right PCA Pulsatility Index | 1.06 (0.4325) | 0.835 (0.4225) | -0.52 | 0.60 |
| Left PCA Pulsatility _Index | 0.87 (0.4825) | 0.865 (0.4675) | -0.43 | 0.67 |
| Breath Holding Index (BHI) |  |  |  |  |
| Right MCA BHI | -0.695 (1.115) | -0.375 (0.7925) | -01.22 | 0.22 |
| Left MCA BHI | -0.92 (1.24) | -0.14 (1.25) | -0.55 | 0.58 |
| Right PCA BHI | -0.445 (1.28) | -0.85 (1.29) | -0.83 | 0.41 |
| Left PCA BHI | -0.475 (0.67) | -0.38 (0.52) | -0.43 | 0.67 |

**AOO***, age of onset;* **DOI,** duration of illness; **LEDD**, Levodopa equivalent daily dose; **MDS-UPDRS**, Movement Disorder Society—Unified Parkinson’s Disease Rating Scale; **PIGD**  postural instability –gait difficulty ; **H and Y,** Hoehn and Yahr scale for Parkinson*;* **NFOG-Q ;**new freezing of gait questionnaire ; **BBS,** berg balance scale **;TUG ,**time up and go test ;**Max**, maximum; **Min,** minimum ;**10 MWT**, 10 meter walk test**; IPAQ,** international physical activity questionnaire; **NMSS,** non-motor symptoms scale of Parkinson; **PDQ-39,** Parkinson’s disease questionnaire; **MMSE**, mini mental states examination; **MOCA,** Montreal cognitive assessment; **FAB**, Frontal Assessment Battery; **BDI,** Beck depression inventory; **WMS**, Wechsler memory scale; **MRI,** Magnetic Resonance Imaging; **HBA1C,** glycated hemoglobin; **LDL**, Low density lipoprotein; **HDL**, High density lipoprotein; **CCA,** common carotid artery; **IMT,** intima media thickness; **PSV,** peak systolic velocity; **ICA,** internal carotid artery; **MCA,** middle cerebral artery; **MVF,** mean flow velocity**; PCA ,**posterior cerebral artery; **BHI,** breath holding index.

*^* *Chi-square test is used *p-value is significant***.***ꭝ Mann Whitney U test was used^*

**Supplementary Table 2. Differences in laboratory tests between vascular parkinsonism and Parkinson’s disease**

| **Laboratory results** | **Parkinson’ disease**  **(No.= 48)** | **Vascular parkinsonism**  **(No. =27)** | **Mann Whitney U test/ T test^** |
| --- | --- | --- | --- |
| Hemoglobin | 13.85 (10.33-16.30) | 12.80 (9.30-10.10) | **0.005*** |
| Albumin | 4 (3.2-5.6) | 3.70 (3.2-4.1) | **<0.001*** |
| HbA1c % | 5.85 (4.40-9.80) | 5.30 (4.50-10.10) | **0.014*** |
| Uric acid (mg/dL) | 5.90 (4.4-7) | 6.60 (2.9-9) | **0.015*** |
| Cholesterol (mg/dL) | 174 (95-313) | 210 (115-351) | **0.002*** |
| Triglycerides (mg/dL) | 111 (60-294) | 111 (57-192) | 0.631 |
| LDL (mg/dL) | 109 (48.0-181) | 118 (59.4-245) | **0.012*** |
| HDL (mg/dL) | 37 (29-66) | 40 (25-77) | 0.156 |

**HbA1c C**, HemoglobinA1C; **LDL**, low-density lipoprotein; **HDL,** high-density lipoprotein.

*^ T-test was used *p-value is significant.*

**Supplementary Table 3: Brain MRI of patients with vascular parkinsonism versus Parkinson’s disease**

|  | **Parkinson’s disease**  **(No. =48)** | **Vascular Parkinsonism**  **(Number =27)** | **Mann Whitney U test** | |
| --- | --- | --- | --- | --- |
|  | Median (Range) | Median (Range) | **z** | **P** |
| Fazekas scale Total | 1 (0 – 2) | 2 (1 – 3) | -6.475 | **<0.001*** |
| Scheltens’ scale | | | | |
| Scheltens’ total score | 1 (0 – 12) | 12 (2 – 22) | 1 (0 – 12) | **<0.001*** |
| Periventricular | 0.5 (0 – 6) | 6 (2 – 6) | 0.5 (0 – 6) | **<0.001*** |
| Deep White Matter | 1 (0 – 6) | 4 (0 – 6) | 1 (0 – 6) | **<0.001*** |
| Basal Ganglion | 0 (0 – 1) | 1 (0 – 4) | 0 (0 – 1) | **<0.001*** |
| Infratentorial | 0 (0 – 1) | 0 (0 – 6) | 0 (0 – 1) | **0.004*** |
|  | | | | |
| Visual rating scale for Atrophy total score | 0 (0 – 2) | 0 (0 –3) | -3.887 | **<0.001*** |

*^* *Chi-square test is used*

**p-value is significant*
